# Epigenetic inactivation of the candidate tumor suppressor gene ASC/TMS1 in human renal cell carcinoma and its role as a potential therapeutic target

**DOI:** 10.18632/oncotarget.4256

**Published:** 2015-06-05

**Authors:** Qianling Liu, Jie Jin, Jianming Ying, Yun Cui, Mengkui Sun, Lian Zhang, Yu Fan, Ben Xu, Qian Zhang

**Affiliations:** ^1^ Department of Urology, Peking University First Hospital and Institute of Urology, National Research Center for Genitourinary Oncology, Beijing 100034, China; ^2^ Department of Pathology, Cancer Institute and Cancer Hospital, Peking Union Medical College (PUMC), Chinese Academy of Medical Sciences, Beijing 100021, China

**Keywords:** ASC/TMS1, tumor suppressor, DNA methylation, renal cell carcinoma, chemosensitivity

## Abstract

This study investigated the epigenetic alteration and biological function of the pro-apoptotic gene ASC/TMS1 in renal cell carcinoma. ASC/TMS1 was downregulated in five out of six RCC cell lines. A significant downregulation was also detected in sixty-seven paired renal tumors compared with adjacent non-cancerous tissues. The downregulation of ASC/TMS1 was correlated with promoter hypermethylation and could be restored with demethylation treatment. Re-expression of ASC/TMS1 in silenced RCC cell lines inhibited cell viability, colony formation, arrested cell cycle, induced apoptosis, suppressed cell invasion and repressed tumorigenicity in SCID mice. The antitumorigenic function of ASC/TMS1 in renal cancer was partially regulated by activation of p53 and p21 signaling. In addition, restoration of ASC/TMS1 sensitizes RCC cells to DNA damaging agents. Knockdown of ASC/TMS1 reduced DNA damaging agents-induced p53 activation and cell apoptosis. Moreover, ASC/TMS1 hypermethylation was further detected in 41.1% (83/202) of RCC tumors, but only 12% in adjacent non-cancerous tissues. ASC/TMS1 methylation was significantly correlated with higher tumor nuclear grade. In conclusion, ASC/TMS1 is a novel functional tumor suppressor in renal carcinogenesis. ASC/TMS1 tumor specific methylation may be a useful biomarker for designing improved diagnostic and therapeutic strategies for RCC.

## INTRODUCTION

Renal cell carcinoma (RCC) is the most lethal type of urological cancers due to its occult onset and resistance to radiation and chemotherapy, accounting for approximately 3% of all adult malignancies [1]. However, the biological and molecular mechanisms underlying renal cancer development remain largely unclear. As an alternative to genetic changes, hypermethylation of CpG rich promoter regions leading to consequent silencing or downregulation of tumor suppressor genes s is now recognized as an important mechanism for renal cancer initiation and progression [2–5]. In addition to its biological relevance to malignant transformation, DNA methylation is also one of the most promising biomarkers for early detection and prognosis assessments of human cancers. Therefore, the identification of novel tumor suppressor genes silenced by promoter hypermethylation may provide a new insight to understand the molecular mechanisms of renal cancer and to determine potential diagnostic and therapeutic target for renal cancer.

Genetic and epigenetic modifications of apoptotic genes was previously reported to affect the development, inflammatory response, carcinogenesis, tumor growth, and chemoradiosensitivity. TMS1 (target of methylation induced silencing), also known as ASC (apoptosis speck-like protein containing a CARD) has been described in the literature as a pro-apoptotic gene which encodes a 22 kDa protein containing a pyrin domain (PYD) in the N-terminus and a caspase recruitment domain (CARD) in the C-terminus. Both PYD and CARD are members of the 6-helix bundle death domain-fold superfamily and are involved in the regulation of apoptosis, nuclear factor-κB activation, and the maturation of proinflammatory cytokines [6]. ASC/TMS1, localized on chromosome 16p11.2–12.1, was identified independently by Masumoto et al. in 1999 while searching for proteins involved in differentiation [7] and Conway et al. in 2000 by screening for targets of methylation-associated gene silencing [8]. Previous studies have shown that the overexpression of ASC/TMS1 induced caspase- 9-dependent apoptosis [9]. However, many other researches have also reported that ASC/TMS1 triggers caspase-8-dependent apoptotic cell death [10]. Futhermore, restoration of ASC/TMS1 expression sensitized colorectal cancer cells to genotoxic stress-induced caspase-independent cell death [11]. The knockdown of ASC/TMS1 reduced sensitivity of cancer cells to cytotoxic agents [7]. All the data above indicated the critical role of ASC/TMS1 in apoptosis and resistance to chemotherapy. Earlier reports have also reported that ASC/TMS1 acts as a tumor suppressor gene and is silenced by DNA promoter hypermethylation in several human carcinomas such as breast, prostate, and ovary cancer [8, 12, 13]. However, the role and the clinical implication of ASC/TMS1 in renal cancer remain unclear. In this study, we examined the epigenetic regulation, biological function, molecular basis of ASC/TMS1 in renal cancer and its potential role in chemotherapy-resistance.

## RESULTS

### Methylation of the ASC/TMS1 promoter correlates with its transcriptional silencing

**Figure 1:**
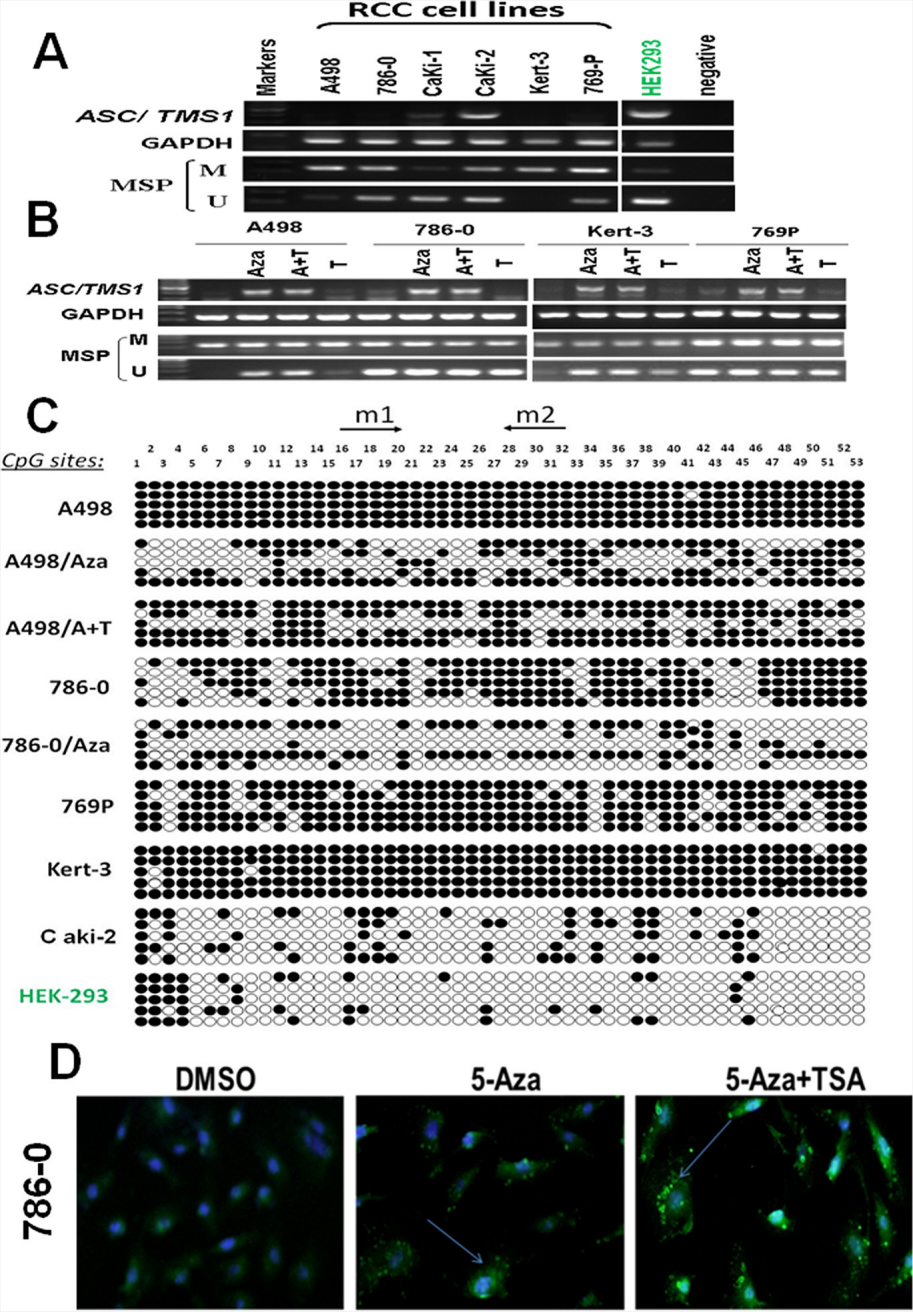
ASC/TMS1 inactivation by promoter hypermethylation in RCC cell lines **A.** ASC/TMS1 was frequently silenced or reduced in RCC cell lines by promoter hypermethylation. HEK293, normal human embryonic kidney cell line. M, methylated. U, unmethylated. **B.** Pharmalogic demethylation with 5-Aza alone or combined with trichostatin A (A + T) restored ASC/TMS1 mRNA expression and induced its demethyation in RCC cell lines. **C.** Methylation status of individual CpG sites in the ASC/TMS1 promoter was confirmed by bisulfite genomic sequencing. Each row represents one bacterial clone with one circle symbolizing one CpG site. Filled ovals indicate methylated. Open ovals indicate unmethylated. **D.** Immunofluorescence staining of ASC/TMS1 protein in 786-O cells. Pharmalogic demethylation with 5-Aza alone or combined with trichostatin A (A + T) restored ASC/TMS1 protein expression in 786-O cells. Green pellet in the cytoplasm and nucleus represents positive staining (indicated by arrows).

By semiquantitative RT-PCR, we first analyzed the mRNA expression status of ASC/TMS1 in six RCC cell lines, which showed that ASC/TMS1 was silenced or downregulated in five out of six (83.3%) renal cancer cell lines (Figure 1A). However, ASC/TMS1 was highly expressed in normal human embryonic kidney cell line (HEK293) (Figure 1A). The region spanning the putative promoter and exon 1 of ASC/TMS1 is a typical CpG island and, thus, susceptible to epigenetic silencing. Therefore, we next explored the role of promoter hypermethylation in silencing ASC/TMS1 by methylation specific PCR. Full or partial methylation was detected in six renal cancer cell lines (A498, 786–0, Caki-1, Caki-2, Kert-3 and 769P), which showed silenced or downregulated ASC/TMS1 expression, whereas only weak methylation was detected in HEK293 with ASC/TMS1 expression (Figure 1A). The methylation density within the ASC/TMS1 promoter region was then characterized and validated by bisulfite genomic sequencing. The bisulfite genomic sequencing results were consistent with those of methylation specific PCR in which dense methylation was found in methylated cell lines (A498, 786–0, Kert-3and 769P), but little in weak methylated HEK293 (Figure 1C). These results showed that ASC/TMS1 downregulation is common in RCC cell lines and closely correlates with its promoter methylation.

### Pharmacological demethylation reactivates ASC/TMS1 expression in RCC cells

To explore whether promoter methylation directly mediates ASC/TMS1 reduction in RCC, four methylated cell lines (A498, 786-O, Kert-3, 769P) were treated with the DNA methyltransferase inhibitor 5-Aza-2′-deoxycytidine with or without the histone deacetylase inhibitor Trichostatin A. After treatment, ASC/TMS1 expression was significantly increased in these cell lines along with an increase in unmethylated promoter alleles and a decrease in methylated alleles (Figure 1B). Further detailed BGS methylation analysis for A498 and 786-O before and after Aza treatment confirmed its demethylation (Figure 1C). To check if demethylation treatment also restored ASC/TMS1 protein level, we performed an immunofluorescence assay using antibody against ASC/TMS1 in 786–0 cells. As shown in Figure 1D, there was a significant increase in the ASC/TMS1 green immunofluorescence signal after 5-Aza and 5-Aza-TSA treatments as compared to DMSO control, suggesting that 5-Aza and 5-Aza-TSA can restore the ASC/TMS1 protein expression in 786–0 cells. These results indicated that CpG hypermethylation of the ASC/TMS1 promoter directly led to its silencing in RCC cell lines.

### ASC/TMS1 mRNA and protein expression was frequently downregulated or silenced in primary RCC tumor tissues

**Figure 2:**
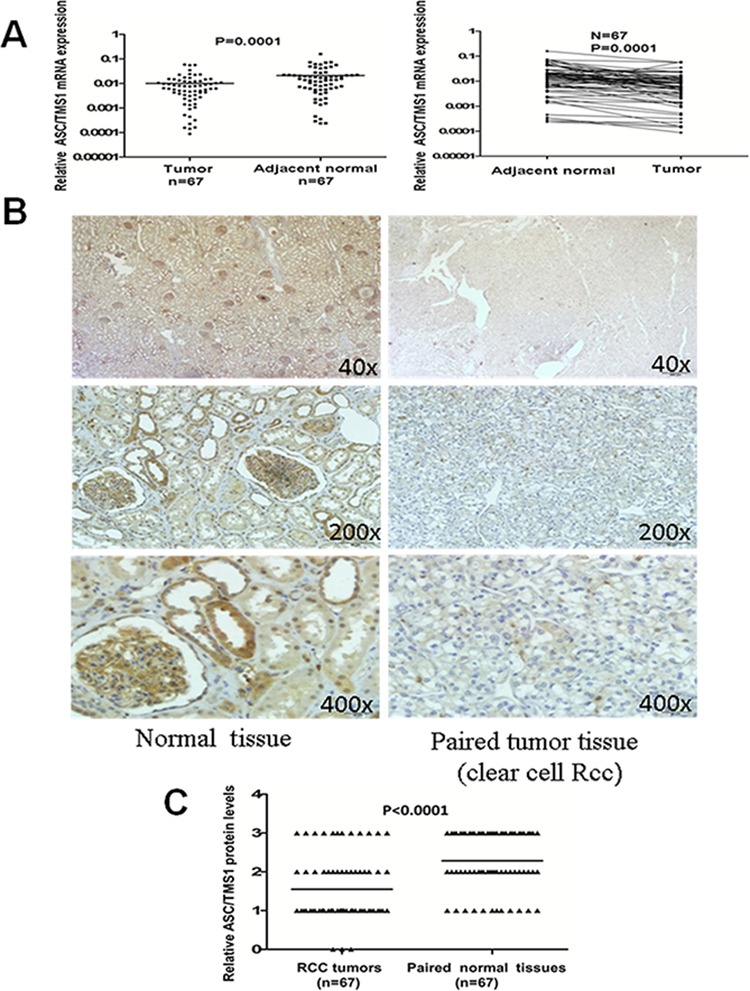
Expression pattern of ASC/TMS1 in RCC **A.** The mRNA expression levels of ASC/TMS1 in paired primary RCC tissues as determined by quantitative real-time PCR. ASC/TMS1 mRNA was significantly downregulated in RCC samples compared with their adjacent normal tissues (*p* = 0.0001). **B.** Representative immunohistochemical staining of a pair of RCC specimens and corresponding nontumor tissues. In adjacent nontumor tissues, intense immunostaining for ASC/TMS1 was detected in a cytoplasmic and nuclear distribution, whereas absent/weak immunostaining was observed in the cytoplasm and nucleus of tumor tissues. **C.** Evaluation and statistical analysis of ASC/TMS1 protein expression in 67 paired primary RCC tissues. ASC/TMS1 protein expression was significantly downregulated in RCC samples compared with adjacent normal tissues (*P* < 0.0001).

The mRNA expression of ASC/TMS1 was evaluated in sixty-seven paired primary RCCs using quantitative Real-Time PCR. As shown in Figure 2A, ASC/TMS1 mRNA was significantly downregulated in renal tumors compared with their adjacent nontumor tissues (*P* = 0.0001). Inhibition of ASC/TMS1 mRNA expression in the carcinoma tissues of renal cancer patients was further confirmed at protein level by using immunohistochemical staining. We examined ASC/TMS1 protein expression in 67 paired primary RCCs. In adjacent nontumor tissues, intense immunostaining for ASC/TMS1 was observed in a cytoplasmic and nucleus distribution (Figure 2B), whereas absent/weak immunostaining was detected in tumor tissues (Figure 2B). Statistical analysis of the immunohistochemical results revealed that protein expression of ASC/TMS1 in RCC tumor tissues was significantly lower than in adjacent nontumor tissues (Figure 2C, *P* < 0.0001).

### Frequent ASC/TMS1 promoter hypermethylation in primary RCC tumors is associated with patient poor prognosis

**Figure 3:**
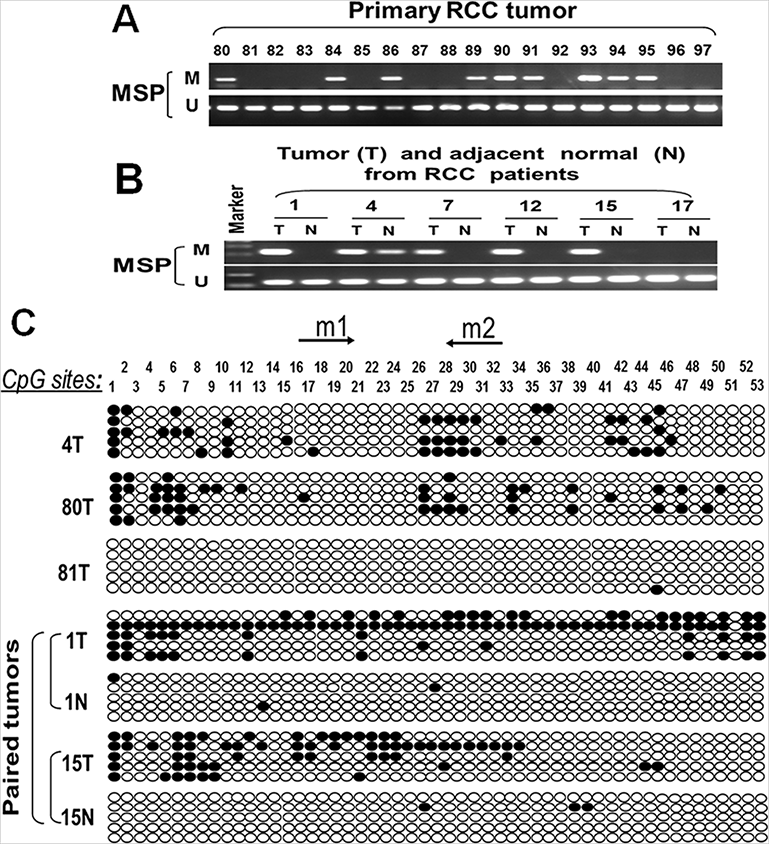
Representative MSP and BGS results **A.** ASC/TMS1 methylation in primary RCC. M, methylated; U, unmethylated. **B.** ASC/TMS1 methylation in paired RCC (T) and matched normal renal tissue (N) samples. **C.** Methylation status of ASC/TMS1 was confirmed by bisulfite genomic sequencing (BGS). Each row represents one bacterial clone with one circle symbolizing one CpG site. Filled ovals indicate methylated. Open ovals indicate unmethylated.

We further analyzed ASC/TMS1 methylation status in paired primary RCC samples and their adjacent nontumor tissues. Of 202 tumor samples 83 (41.1%) showed methylation, but only 12% (3/25) in adjacent non-malignant renal tissues, suggesting tumor-specific methylation of ASC/TMS1 in RCC. Representative methylation status of ASC/TMS1 in RCC primary tumors (T) and paired adjacent nontumor tissues (N) are shown in Figure 3A and 3B. MSP results was confirmed by bisulfite genomic sequencing (Figure 3C). The relationship of ASC/TMS1 methylation with the clinicopathological features of these patients was also analyzed. As shown in Table 1, there was a significant correlation between ASC/TMS1 methylation and tumor nuclear grade of RCC (*p* = 0.005), whereas no significant correlation was found between its methylation and gender, age, tumor location, TNM stage and histological type. These data indicate that ASC/TMS1 methylation is a frequent event in pathogenesis of RCC and is associated with patient poor prognosis.

**Table 1:** Association between ASC/TMS1 methylation and clinicopathological features of patients with RCC

Clinicopathological features	Number (*n* = 202)	ASC/TMS1 methylation status		*P* Value
		Methylated	Umethylated	
Overall	202	83(41.1)	119(58.9)	
Gender:				
M	138	59(42.8)	79(57.2)	0.540
F	64	24(37.5)	40(62.5)	(Fisher's exact test)
Age:				
< 60(median)	106	44(41.5)	62(58.5)	1.000
≥ 60	96	39(40.6)	57(59.4)	(Fisher's exact test)
Side:				
Rt	109	45(41.3)	64(58.7)	1.000
Lt	93	38(40.9)	55(59.1)	(Fisher's exact test)
TNM classification:				
pT1a	77	26(33.8)	51(66.2)	0.428
pT1b	68	31(45.6)	37(54.4)	(chi-square test)
pT2	18	8(44.4)	10(55.6)	
pT3	39	18(46.2)	21(53.8)	
Nuclear grade:				
G1	49	14(28.6)	35(71.4)	0.005*
G2	128	51(39.8)	77(60.2)	(chi-square test)
G3	25	17(68.0)	8(32.0)	
Histological type:				
Clear cell Rcc	185	79(42.7)	106(57.3)	0.305
papillary Rcc	9	2(22.2)	7(77.8)	(chi-square test)
chromophobe Rcc	8	2(25.0)	6(75.0)	

### ASC/TMS1 inhibits renal cancer cell growth

**Figure 4:**
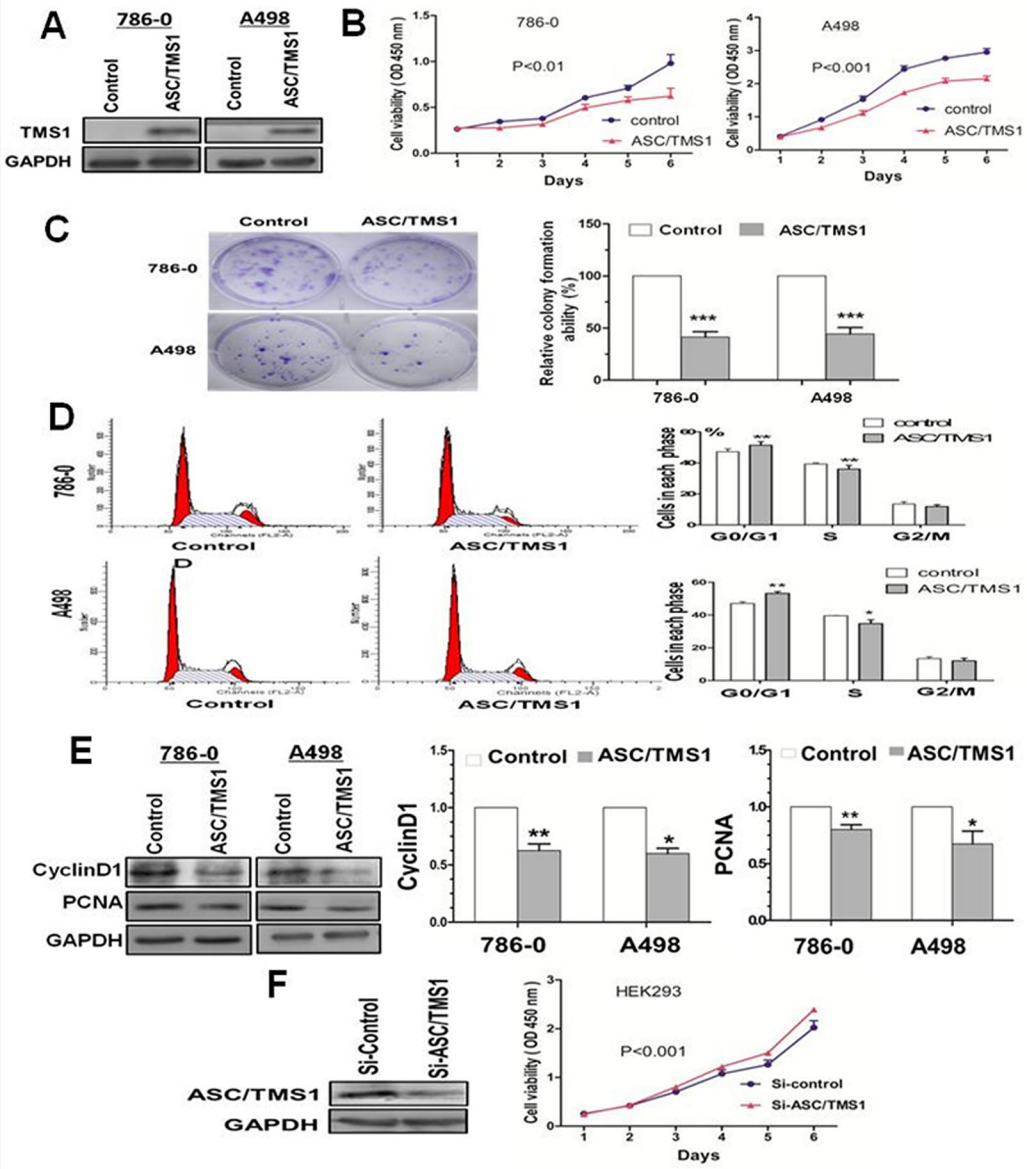
Effect of ectopic ASC/TMS1 expression on tumor growth **A.** Ectopic expression of ASC/TMS1 protein was confirmed by western blot. **B.** Cell growth curve was inhibited by ASC/TMS1 in 786–0 and A498 cells. **C.** ASC/TMS1 suppressed colony formation in 786–0 and A498 cells. **D.** ASC/TMS1 causes cell cycle arrest in G0/G1 phase. **E.** Re-expression of ASC/TMS1 suppresed the protein expression of CyclinD1 and PCNA in 786–0 and A498 cells. **F.** ASC/TMS1 knockdown by si-ASC/TMS1 increased cell growth ability in a normal HEK293. The data are means ± s.d. of three separate experiments. *P < 0.05; **P < 0.01; and ***P < 0.001.

The frequent silencing of ASC/TMS1 mediated by promoter hypermethylation in RCC, but not in normal renal tissue, suggested that ASC/TMS1 may be a candidate tumor suppressor in renal carcinogenesis. We thus examined the growth inhibitory effect through ectopic expression of ASC /TMS1 in silenced renal cancer cell lines 786–0 and A498. Restored expression of ASC/TMS1 was evidenced by western blot (Figure 4A), which dramatically suppressed cell growth curve in both the cell lines (Figure 4B). The inhibitory effect on cell growth was further confirmed by colony formation assay that ASC/TMS1 inhibited the number of colonies in 786–0 and A498 (Figure 4C). Moreover, ASC/TMS1 reduced protein expression of proliferating cell nuclear antigen, a marker of cell proliferation (Figure 4E).

### ASC/TMS1 causes cell cycle arrest in G0/G1 phase

We investigated the effects of ASC/TMS1 on cell cycle distribution. Flow cytometry analysis of ASC/TMS1-transfected 786–0 and A498 revealed a significant decrease in the number of cells in the S phase compared with controls (Figure 4D), conferring the inhibitory effect of ASC/TMS1 on cell proliferation. Concomitant with this inhibition, there was a significant increase in the number of cells accumulating in the G0/G1 phase (Figure 4D), thus ASC/TMS1 blocks the cell cycle at the G0/G1 checkpoint. In addition, Our results showed that a key G1 phase regulator cyclin D1 was downregulated in ASC/TMS1-transfected 786–0 and A498 as compared with the vector-transfected controls (Figure 4E).

### ASC/TMS1 inhibits RCC cell migration and invasion

**Figure 5:**
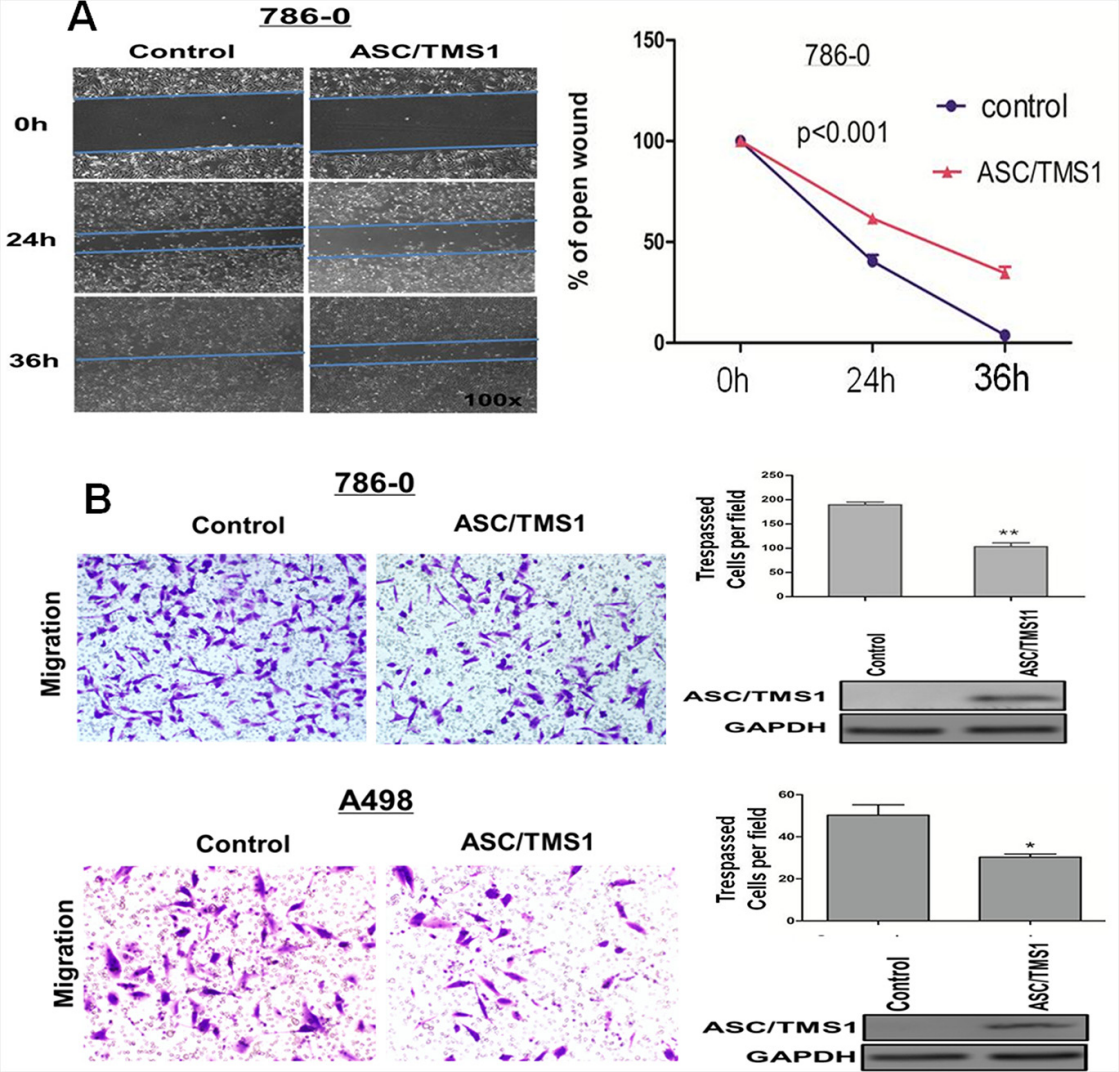
ASC/TMS1 modulates RCC cell migration and invasion **A.** The migration of 786–0 cells in wound healing experiment. Ectopically expressed ASC/TMS1 suppressed RCC cell migration in 786-O cells. Photographs were taken at 0, 24 and 36 h to determine the different mobility between 786–0/control and 786–0/ASC/TMS1. **B.** Cell invasion of 786–0 and A498 cells in matrigel assay. ASC/TMS1 suppressed RCC cell invasion in 786–0 and A498 cells. The data are means ± s.d. of three separate experiments. *P < 0.05; and **P < 0.01.

To investigate the effect of ASC/TMS1 on RCC cell migration, the monolayer wound-healing assay was performed. A significant delay in the closure of the wound gaps in 786–0 cells transfected with ASC/TMS1 as compared with cells transfected with empty vector was observed at both 24 and 36 h (Figure 5A). For the quantitative assessment of cell metastasis and invasiveness, we performed the matrigel invasion assay. The invaded cell number in 786–0 and A498 with ASC/TMS1 expression was significantly lower than in control 786–0 and A498 without ASC/TMS1 expression (Figure 5B), suggesting that ASC/TMS1 inhibits the migration and the invasion of RCC cells.

### ASC/TMS1 induces cell apoptosis and plays a role in activation of p53

**Figure 6:**
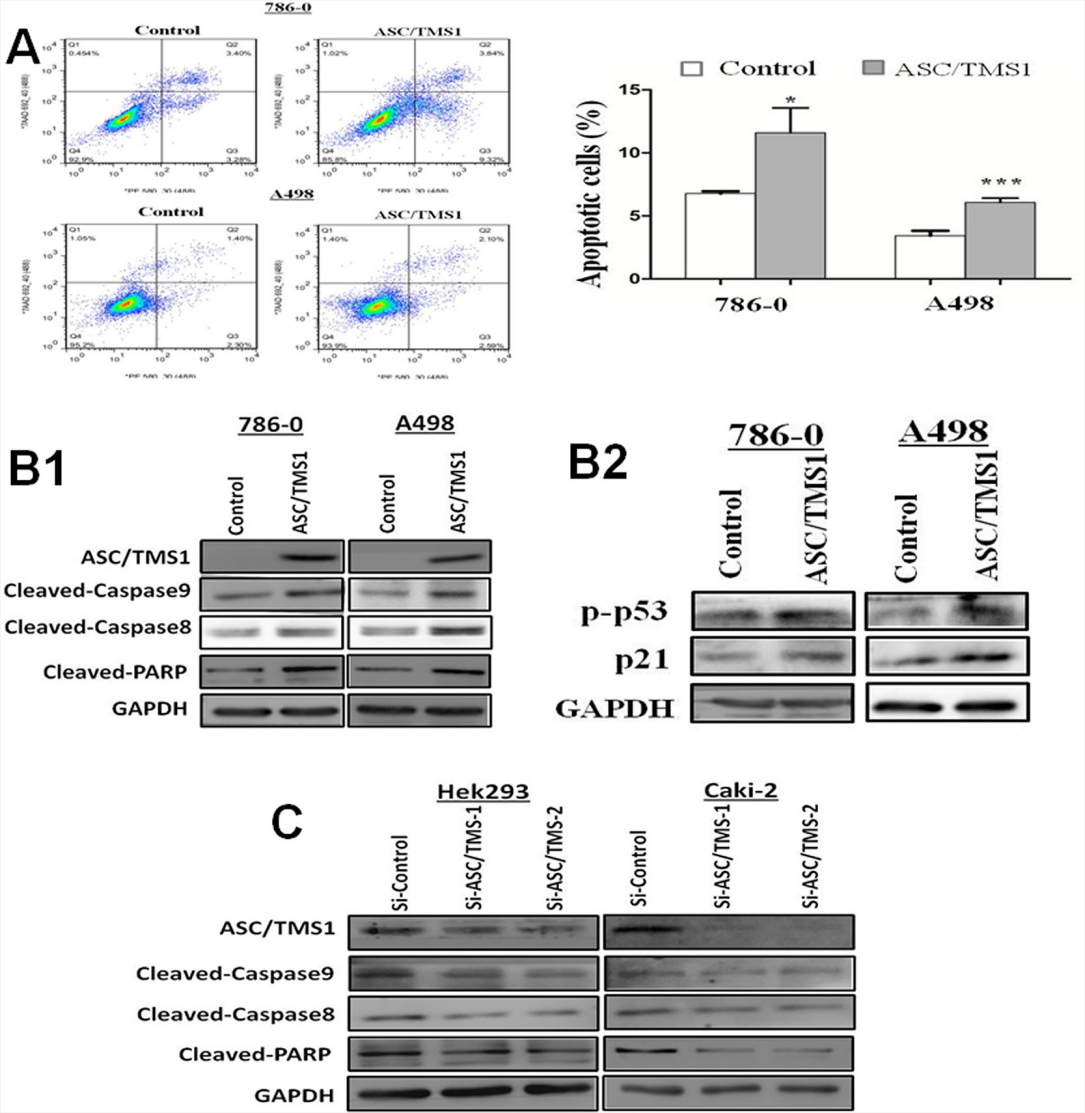
ASC/TMS1 induced apoptosis of renal cancer cells **A.** Flow cytometry assay with Annexin V:PE and 7AAD double staining. **B1.** Overexpression of ASC/TMS1 induced the protein expression of cleaved caspase-8, cleaved caspase-9, cleaved PARP in 786–0 and A498 cells by western blot. **B2.** Ectopic expression of ASC/TMS1 enhanced the protein expression of p-p53 and p21 using western blot. GAPDH was used as an internal control. **C.** Knockdown of ASC/TMS1 reduced the protein expression of cleaved caspase-8, cleaved caspase-9, cleaved PARP in 786–0 and A498 cells by western blot. *P < 0.05; and ***P < 0.001.

To determine whether the ASC/TMS1-mediated growth inhibition was the result of apoptosis, we performed an apoptosis assay using Annexin V:PE (BD Biosciences) and 7-amino-actinomycin (BD Biosciences) double staining. Ectopic expression of ASC/TMS1 resulted in a significant increase in apoptotic cells as compared with vector control in 786–0 cells (Figure 6A). In addition, induction of apoptosis was further assessed by immunoblot detection of p-p53 and its downstream target genes p21, the active form of caspase-8, caspase-9, and poly (ADP-ribose) polymerase (PARP). As shown in Figure 6B1 and 6B2, overexpression of ASC/TMS1 enhanced the levels of active form of p53, p21, caspase -8, caspase-9, and PARP.

### Knockdown of ASC/TMS1 promotes cell growth and reduces cell apoptosis

To further confirm the role of ASC/TMS1 in cell growth and apoptosis, the effect of ASC/TMS1 was investigated through knockdown ASC/TMS1 with small interfering RNA (siRNA) in normal human embryonic kidney cell line HEK293 and RCC cell line Caki-2. Reduced expression of ASC/TMS1 was evidenced by western blot (Figures 4F and 6C). Knockdown of ASC/TMS1 in HEK293 markedly enhanced cell viability (Figure 4F) compared to cells treated with the control siRNA. On the other hand, small interfering RNA (siRNA) mediated knockdown of ASC/TMS1 in HEK293 and Caki-2 reduced the expression levels of active caspase -8, caspase -9, and PARP (Figure 6C). These data provide evidences that ASC/TMS1 functions as a potential tumor suppressor in RCC.

### ASC/TMS1 inhibits tumor growth in SCID mice

**Figure 7:**
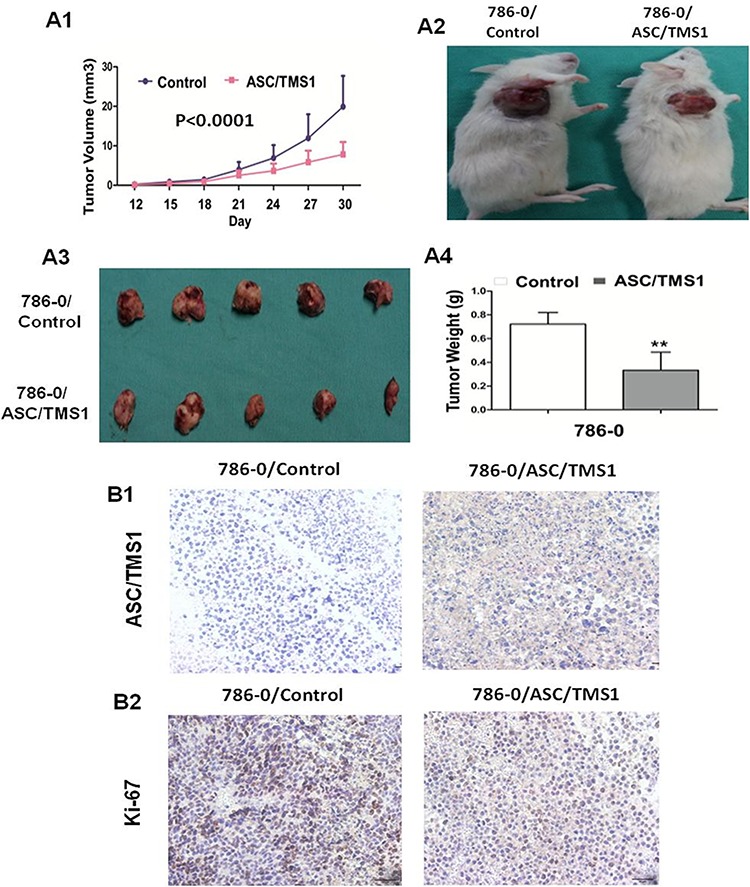
ASC/TMS1 inhibited growth of tumors derived from 786–0 *in vivo* **A1.** Subcutaneous tumor growth curve of ASC/TMS1-expressing 786–0 cells in SCID mice was compared with control empty vector transfected cells. **A2.** A representative picture of tumor growth in SCID mice subcutaneously inoculated with ASC/TMS1 or control vector. **A3.** Pictures of the isolated tumors from each mouse (*n* = 5/group). **A4.** Histogram represents mean of the tumor weight from the ASC/TMS1 and control vector groups. Data are mean ±SD. **B1.** Immunohistochemical staining of ASC/TMS1 in xenograft tumors in SCID mice. Brown cytoplasmic and nuclear signals indicate the ASC/TMS1 protein expression. **B2.** Representative ki-67 staining of xenografted tumor derived from 786–0 cells transfected with ASC/TMS1 or control vector. An decrease in the number of Ki-67-positive cells (brown-stained nuclei) is evident in ASC/TMS1-transfected tumors. **P < 0.01.

In light of the observed anti-proliferative and pro-apoptotic effects of ASC/TMS1 on the cell lines *in vitro*, we tested whether ASC/TMS1 could alter growth of RCC cells *in vivo*. The tumor growth of 786–0 stably transfected with ASC/TMS1 or empty vector in SCID mice was shown in Figure 7A. The tumor growth was significantly lower in ASC/TMS1-transfected SCID mice as compared with the vector control mice (*P* < 0.0001; Figure 7A1). Moreover, the mean tumor weight and staining intensity of proliferation antigen Ki-67 were significantly lesser in ASC/TMS1-transfected tumors compared with the control vector transfected tumors (Figure 7A4 and 7B2), inferring that ASC/TMS1 does function as a tumor suppressor in renal carcinogenesis. Expression of ASC/TMS1 protein in isolated tumor cells transfected with ASC/TMS1 was confirmed with ASC/TMS1-positive signal in cytoplasm by immunohistochemistry (Figure 7B1). This suggests that ASC/TMS1 plays an important role in preventing cell overgrowth *in vivo*.

### ASC/TMS1 expression sensitizes human renal cancer cells to DNA damaging agents

**Figure 8:**
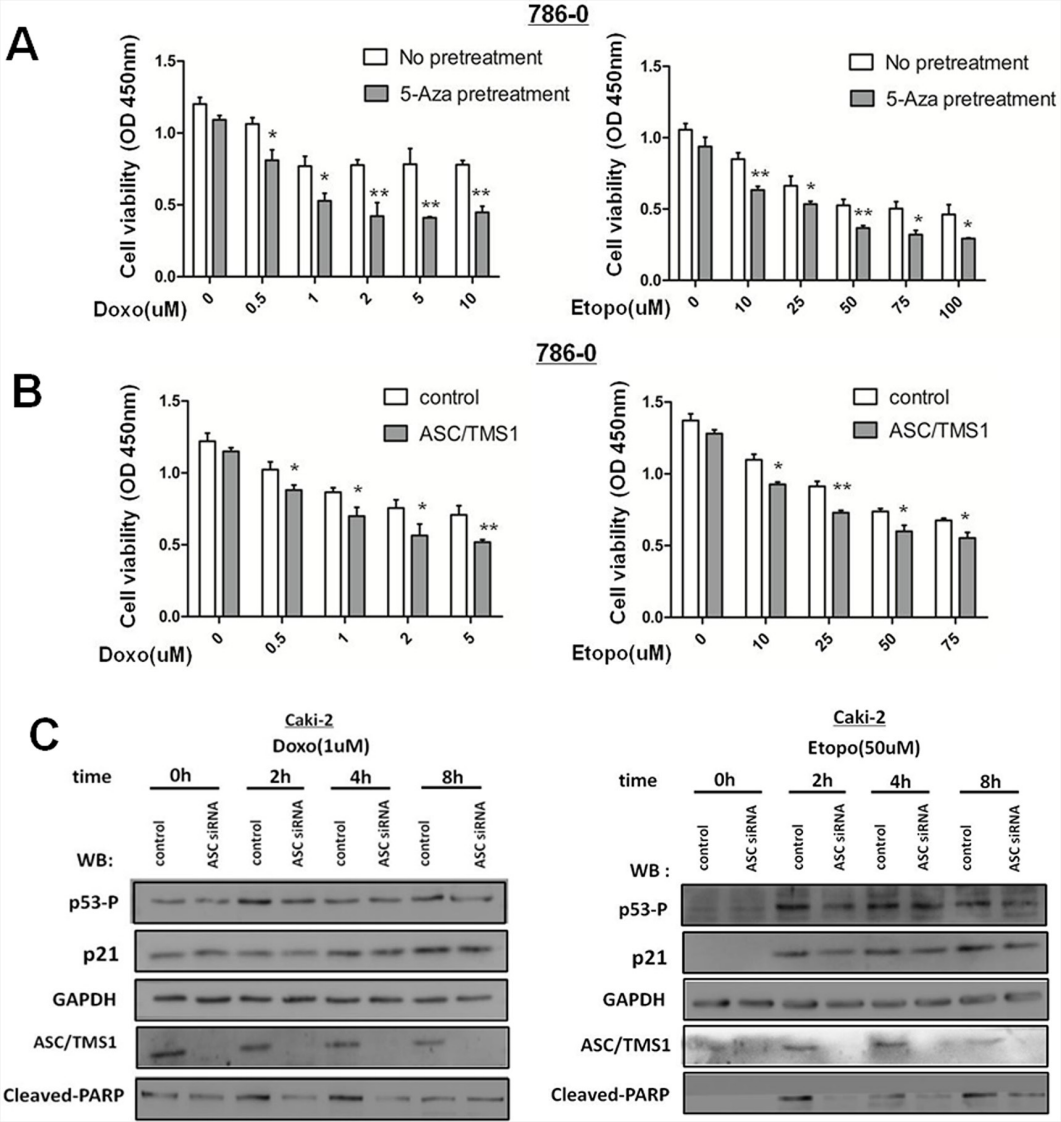
ASC/TMS1 expression sensitizes human renal cancer cells to DNA damaging agents **A.** 786–0 cells with or without 5-Aza-priming (10 μm, 72 h) were treated with etoposide or doxorubicin at the indicated concentration for additional 36 h. 5-Aza-primed 786–0 cells showed a significant decrease in cell survival compared with unprimed 786–0 cells, as revealed by the CCK8 assay. **B.** 786–0-control or 786–0-ASC/TMS1 cells were treated with etoposide or doxorubicin at the indicated concentration for 36 h. Cell death was significantly higher in the 786–0-ASC/TMS1 cells than that in the control 786–0 cells, as revealed by the CCK8 assay. **C.** Caki-2 cells were transfected with si-control or si-ASC/TMS1 RNA (50 nM), and after 48 h of transfection, cells were treated with etoposide (50 uM) or doxorubicin (1 uM) and analyzed by Western blot for the indicated antibodies.

As RCC are resistance to radiation and chemotherapy, therefore we explored whether restoration of pro-apoptotic gene ASC/TMS1 might sensitizes human renal cancer cells to DNA damaging agents. As a result, up-regulation of ASC/TMS1 by pre-treatment with 5-Aza or recombinant expression in human renal cancer 786–0 cells, in which ASC/TMS1 is silenced by aberrant methylation, potentiated cell death mediated by DNA damaging agent etoposide and doxorubicin [Figure 8A and 8B].

### Decreased ASC/TMS1 reduces DNA damaging agents-induced p53 activation and cell apoptosis in renal cancer Caki-2 cells

To verify the above-mentioned observation that ASC/TMS1 expression sensitizes renal cancer cells to DNA damaging agents, we knockdowned ASC/TMS1 expression in human renal cancer Caki-2 cells, in which ASC/TMS1 is highly expressed. ASC/TMS1 expression in Caki-2 cells was markedly decreased by transfection with a siRNA specific to ASC/TMS1 (Figure 8C). As it has demonstrated that loss of ASC/TMS1 correlates with reduced UVB-induced p53 activation in murine skin previously, so we explored whether loss of ASC/TMS1 might reduce p53 activation in renal cancer Caki-2 cells following treatment with DNA damaging agents etoposide and doxorubicin, two known inducer of p53 activation. As a result, knockdown for ASC/TMS1 with a specific siRNA resulted in reduced p53 phosphorylation at serine 15 and p21 expression (Figure 8C). In addition, PARP cleavage were attenuated in ASC/TMS1-knockdowned Caki-2 cells (Figure 8C). Taken together, these results reinforce the conclusion that ASC/TMS1 plays a role in p53 activation, and further indicates that ASC/TMS1can refrain proliferation and promote apoptosis of renal cancer cells.

## DISCUSSION

In this study we found that ASC/TMS1 mRNA is widely expressed in normal adult renal tissues and human embryonic kidney cell line HEK293, whereas frequently silenced or downregulated in RCC cell lines and primary RCC tumors. This finding was further confirmed by immunohistochemistry, where a remarkable loss of ASC/TMS1 immunostaining in most studied primary renal tumors was contrasted by a strong, diffuse cytoplasmic and nucleus staining in adjacent non-malignant renal tissues, as has been observed in other normal tissues [14]. Regrettably, the sixty-seven pairs of specimens used for mRNA expression and protein expression are mismatching. So we can't further analysis the association between mRNA expression and protein expression of ASC/TMS1. The silencing or downregulation of ASC/TMS1 was revealed to be correlated with promoter hypermethylation, as demonstrated by methylation specific PCR and bisulfate genomic sequencing analysis. Furthermore, re-expression accompanied by partial demethylation of ASC/TMS1 following 5-Aza-2′-deoxycytidine confirms that promoter hypermethylation of ASC/TMS1 directly mediated its transcriptional silence in RCC. Lack of response to TSA treatment alone suggests that histone acetylation is not the main cause of ASC/TMS1 inactivation in RCC. This finding is similar to the results obtained by Partha M Das and Stimson et al [12, 15]. Of note, we observed promoter hypermethylation and mRNA expression of ASC/TMS1 in Caki-2 cell line. This same discovery that ASC/TMS1 methylation does not always correspond to a lack of ASC/TMS1 expression, has also been observed in a previous report [16]. One possible explanation for this is hypermethylation only to a certain extent (>60%) can lead to gene inactivation [17], or maybe there exists another mechanism for the expression of ASC/TMS1, despite the methylation of its promoter region.

ASC/TMS1 gene inactivation in RCC cell lines and tumor tissues, but expression in normal renal tissues and human embryonic kidney cell line suggested that ASC/TMS1 could be a potential tumor suppressor and its downregulation could have functional role in the development of renal cancer. Therefore, the biological function of ASC/TMS1 in RCC was further investigated by gain- and loss-ASC/TMS1 function assays. Ectopic expression of ASC/TMS1 in the silenced A498 and 786–0 cells significantly inhibited cell proliferation and arrested cell cycle at G0/G1 phase. Concomitantly, ectopic expression of ASC/TMS1 significantly decreased cell migration and cell invasion ability in RCC cell lines [Figure 5A and 5B]. Conversely, siRNA-mediated knockdown of ASC/TMS1 in normal human embryonic kidney cell line HEK293 significantly promoted the cell viability and proliferation [Figure 4F]. Having observed the substantial suppression of RCC cell growth by ASC/TMS1 *in vitro*, we studied tumor suppressive effect of ASC/TMS1 against renal tumor formation *in vivo*. Our result showed that the tumor growth was significantly retarded in SCID mice inoculated with 786–0/ASC/TMS1 compared with those inoculated with 786–0/empty vector subcutaneously. Taken together, the consistent results observed *in vitro* and *in vivo* indicated for the first time that ASC/TMS1 functions as a tumor suppressor in renal carcinogenesis. This understanding of the anti-tumor effect of ASC/TMS1 in RCC suggests that restoration of the function of ASC/TMS1 could halt or reverse the abnormalities, thus having a potential therapeutic effect.

Given these data apparently suggesting that ASC/TMS1 could be a growth inhibitor in renal cancer, thus a better understanding of the mechanism of the action of ASC/TMS1 is required. ASC/TMS1 has been reported as a pro-apoptotic gene which encodes a 22 kDa protein containing a COOH-terminal caspase recruitment domain (CARD) that promotes apoptosis and a NH3-terminal Pyrin domain (PYD) involved with the nuclear factor-κB pathway. Both PYD and CARD domains are members of the death domain superfamily, which mediates assembly of complexes that mediate apoptotic and inflammatory signaling pathways [6]. The cell death-promoting role of ASC/TMS1 has been demonstrated in many reports; However, the exact mechanism through which ASC/TMS1 induces apoptosis is quite controversial depending on the cell type. The most intriguing involvement of ASC/TMS1 in cell death is the mediation of caspase-1 dependent pyroptosis through inflammasome formation [18]; However, pyroptosis is known to mainly happen in innate immune cells to clear intracellular pathogens [19]; In addition to pyroptosis, overexpression of ASC/TMS1 was initially shown to induce apoptosis in a caspase-9 dependent pathway in 293T cells [9]. On the contrary, many other researches have also reported that ASC/TMS1 triggers caspase-8-dependent apoptotic cell death [10, 20, 21].

These reports indicated the critical role of ASC/TMS1 in apoptosis. We thus analyzed the effects of ASC/TMS1 on cell apoptosis in two human RCC cell lines A498 and 786–0. Increased apoptosis was revealed in ASC/TMS1-reexpressed cells by FACScan analysis [Figure 6A]. The induction of apoptosis by ASC/TMS1 was mediated through a caspase-dependent pathway including activation of caspase-8 and caspase-9, two initiator caspases, followed by the proteolytic cleavage of PARP which causes loss of DNA repair, cellular disassembly and finally undergoing apoptosis [22]. These are consistant with the previous studies that overexpression of ASC/TMS1 promotes apoptosis in a caspase 9 dependent pathway [9] and ASC/TMS1 can also promote apoptosis by driving the cleavage of caspase-8, although it is not necessary for caspase-8 activation [10, 23]. Collectively, we guess ASC/TMS1 may promote apoptosis through both the intrinsic mitochondrial and extrinsic death receptor pathways in RCC cell lines.

It has been reported that p53 is a pro-apoptotic gene which can regulates both the intrinsic mitochondrial and extrinsic death receptor apoptotic pathways. Recently, Drexler et al. [24] demonstrated that ASC/TMS1 could display cell-type-specific functions, i.e., it has a proinflammatory role in myeloid cells whereas in epidermal keratinocytes, ASC/TMS1 acts as a tumor suppressor, probably in association with p53 activation. Renal cancers mainly originated from the renal tubular epithelial cells, so we wonder wether ASC/TMS1 has a function in the activation of p53 and its target genes in RCC. We thus further elucidated the molecular basis of ASC/TMS1 exerting its tumor suppressor function in renal cancer by western blot. The enhanced apoptosis ability by ASC/TMS1 was discovered to be mediated at least by activation of pro-apoptotic p53. p53 is known as a tumor suppressor gene which is capable BCL2 of inducing transcriptional regulation of other anti-tumorigenic molecules [25–28]. Induction of p53 had been reported to downregulate BCL2 in breast cancer [29] and upregulate BAX in lung cancer [30], two important mediators in apoptosis process. BAX induces programmed cell death by cytokine deprivation and suppresses activity of BCL2, an apoptotic blocker [30, 31]. Therefore, activation of p53 and its downstream targets induced by ASC/TMS1 could partially explain the effect of ASC/TMS1 in inducing cell apoptosis. Interestingly, it is worth mentioning Ohtsuka et al. [32] demonstrated that BAX protein could bind to the PYD domain of ASC/TMS1. They revealed that ASC/TMS1 acted as a carrier protein of BAX by translocating it to mitochondria. However, a recent study has claimed that the BAX-dependent apoptosis could be linked to the activation of caspase-8 [20]. All in all, further studies are still needed to explore the exact mechanism through which ASC/TMS1 induces apoptosis.

Based on the western blot analyses, the cell cycle arrest in G0/G1 phase caused by ASC/TMS1 was mostly attributed to the upregulation of p21 and downregulation of cyclin D1 (Figure 6B2 and 4E). It has been demonstrated that p53 can directly increase the transcription of p21 [33]. p21 is a potent inhibitor of cyclin D/Cdk4, whose kinases supervise cell cycle progression at the restriction and late transition points of G1 [34]. In addition, downregulation of cyclin D1, a key regulatory subunit of CDK4, could repress the cell cycle entry into S phase [35, 36]. p53 was also known to cause G1 cell cycle arrest by indirectly inhibiting cyclin D1 transcription [37].

Cell migration and invasion are critical events during the cancer progression to metastasis. Re-expression of ASC/TMS1 inhibited the cell migration, as demonstrated by the wound healing assay (Figure 5A). This effect was further varified by matrigel invasion analysis (Figure 5B), suggesting that ASC/TMS1 could reduce invasiveness and metastasis of RCC cells. The possible explanation for the anti-migration/invasion effect caused by ASC/TMS1 in RCC cells maybe contributed to the regulation of NF-κB pathway [6]. However, further studies are needed for us to explore the exact mechanism.

To investigate the clinical application of ASC/TMS1 in renal tumorigenesis, we examined the promoter methylation of ASC/TMS1 by methylations pecific PCR in 202 primary RCCs and 25 normal controls. ASC/TMS1 gene promoter was found to be commonly methylated in RCCs (41.1%, 83/202) as compared with normal controls (12.0%, 3/25). Recognizing the tumor suppressor effect of ASC/TMS1, the inactivation of this gene by promoter hypermethylation would favor tumor progression and a worse outcome. Therefore, the clinical significance of ASC/TMS1 promoter hypermethylation and its associations with patient outcome were evaluated in 202 primary RCC patients. As shown in Table 1, there was a significant correlation between ASC/TMS1 hypermethylation and tumor nuclear grade of RCC (*p* = 0.005), whereas no significant correlation was found between its methylation and gender, age, tumor location, TNM stage and histological type. Previous studies [38] have shown that the hypermethylation of ASC/TMS1 to be a late-stage event in the progression of lung cancer. ASC/TMS1 hypermethylation has also been associated with Stage 4 progressing neuroblastomas [39]. Our study also shows an increased percentage of ASC/TMS1 methylation in Stage 2 or higher tumors compared with Stage 1 patients (although this was not statistically significant). Collectively, these data indicate that ASC/TMS1 methylation is a frequent event in pathogenesis of RCC and is associated with patient poor prognosis. ASC/TMS1 methylation maybe regarded as a potential new prognostic factor for RCC.

Radical nephrectomy is effective to cure local and early RCCs, however, 30% of patients develop metastases after surgery due to its resistance to radiation and chemotherapy. The molecular mechanisms responsible for chemotherapy-resistance in RCC remain unclear. Current studies have reported that in cancer cells, defects in the apoptotic pathway contribute to resistance to chemotherapy [40]. Epigenetic silencing of tumor suppressor and pro-apoptotic genes is one of the mechanisms of resistance of cancer cells to chemotherapeutic drugs [40, 41]. As we know, tumor suppressor gene p53 plays a crucial role in carcinogenesis and chemosensitivity [42]. Our previous work have demonstrated that ASC/TMS1 is a pro-apoptotic gene which plays a role in activation of p53 in RCC. ASC/TMS1 was also identified to be a target of methylation-induced gene silencing in RCC. An inactivation of ASC/TMS1 by promoter hypermethylation might thus cause resistance to chemotherapy in RCC, and if this is the case, then the re-expression of ASC/TMS1 would restore the chemosensitivity and thus become a new treatment strategy for renal cancers. So we explored to clarify this hypothesis.

As a result, up-regulation of ASC/TMS1 by pre-treatment with 5-Aza or recombinant expression in human renal cancer 786–0 cells, in which ASC/TMS1 is silenced by aberrant methylation, potentiated cell death mediated by DNA damaging agent [Figure 8A and 8B]. To verify the above-mentioned observation that ASC/TMS1 expression sensitizes renal cancer cells to DNA damaging agents, we knockdowned ASC/TMS1 expression in human renal cancer Caki-2 cells, in which ASC/TMS1 is highly expressed. As Drexler et al. [24] has previously demonstrated that ASC/TMS1 acts as a tumor suppressor, probably in association with p53 activation. UVB treatment of keratinocytes promoted a transient interaction between ASC/TMS1 and p53. The loss of ASC/TMS1 reduced UVB-induced p53 phosphorylation and subsequently decreased the expression of its target genes. So we wondered whether loss of ASC/TMS1 might reduce p53 activation in renal cancer Caki-2 cells following treatment with DNA damaging agents etoposide and doxorubicin, two known inducer of p53 activation [43]. As a result, knockdown for ASC/TMS1 with a specific siRNA resulted in reduced p53 phosphorylation at serine 15 and p21 expression (Figure 8C). In addition, PARP cleavage were attenuated in ASC/TMS1-knockdowned Caki-2 cells (Figure 8C). Taken together, these results confirm the hypothesis that ASC/TMS1 expression would enhance the chemosensitivity of renal cancer cells, and further indicates that ASC/TMS1 plays a role in p53 activation. The reduced expression of ASC/TMS1 in cancer cells can block apoptotic cell death and contribute to resistance to chemotherapy. Gene introduction of ASC/TMS1 would increase chemosensitivity, and thus become a new treatment strategy for renal cancers.

In conclusion, we have identified a novel functional tumor suppressor gene ASC/TMS1 inactivated by promoter hypermethylation in RCC, with important functions in suppressing cell proliferation, inducing apoptosis and inhibiting cell invasion. ASC/TMS1 plays an important role in activation of p53 and its downstream targets including p21, Cyclin D1, PCNA, active form of caspase-8, caspase-9, and poly (ADP-ribose) polymerase (PARP). ASC/TMS1 tumor specific methylation may serve as a biomarker for early detection and prognosis prediction of RCC, especially when tumor suppressor gene (TSG) hypermethylation can be detected in patients' serum and urine samples, just like RASSF1A, tissue inhibitor of metalloproteinase-3 and CDH1 methylation [44]. Furthermore, the restoration of ASC/TMS1 expression by demethylating agents could be a potent strategy to enhance the efficacy of chemotherapeutic drugs in ASC/TMS1-silenced renal cancer patients.

## MATERIALS AND METHODS

### Patients and tissue samples

All human primary RCCs (202 cases) and adjacent nonmalignant renal tissues were obtained from the urology department of Peking University First Hospital in Beijing from September 2012 to May 2014, with patients'consent according to the university policy. All cases were collected from primary surgical resection with no prior history of RCC and adjuvant therapy. Specimens were snap frozen in liquid nitrogen and subsequently stored at −80°C for molecular analyses. Pathological diagnosis was done and confirmed at the pathology department, Institute of Urology, Peking University First Hospital. The histopathology of tumors was classified by 2009 AJCC TNM stage and Fuhrman nuclear grade.

### Cell culture

Six RCC cell lines (A498, 786-O, Caki-1, Caki-2, Kert-3 and 769P) obtained from American Type Culture Collection and the normal human embryonic kidney cell line HEK293, which serves as a “normal” control for RCC, were routinely maintained in RPMI 1640 or DMEM medium supplemented with 10% fetal bovine serum (GIBCO Invitrogen, Carlsbad, CA, USA) in a humidified incubator at 37°C with an atmosphere of 5% CO2.

### Construction of ASC/TMS1 expression vector and ASC/TMS1 siRNA vector

The expression vector (PEX-2–ASC/TMS1) encoding the full length open reading frame of human ASC/TMS1 gene and the control empty vector PEX-2 were constructed by Su Zhou GenePharma, China. Sequence corresponding to the open reading frame of ASC/TMS1 was amplified by PCR and verified by DNA sequencing. The siRNA specific for ASC/TMS1 and its control siRNA were purchased from OriGene (Rockville, MD, USA).

### 5-Aza-2′-deoxycytidine treatment

For 5-Aza-2′-deoxycytidine (Sigma, St. Louis, MO) and Trichostatin A (TSA) (Sigma St. Louis, MO) treatment, cell lines were grown in a 6-well plate and treated with 10 μM 5-Aza-2′-deoxycytidine for 72 hours and subsequently with or without 100 nM trichostatin A for 24 hours, as described previously [45]. Controlled cells were treated with an equivalent concentration of dimethyl sulfoxide.

### Semiquantitative RT–PCR and real-time quantitative PCR analyses

Total RNA was extracted from RCC cell lines using Trizol reagent (Invitrogen, Carlsbad, CA, USA). For semiquantitative RT-PCR, ASC/TMS1 gene was amplified using AmpliTaq Gold DNA polymerase (Applied Biosystems, Foster City, CA, USA) as reported previously. Real-time PCR was performed using GoTaq(R) qPCR Master Mix (Promega Biotech, Madison, WI, USA) according to the manufacturer's protocol on 7500 Fast Real-Time PCR System (ABI). Primer sequences and PCR conditions are listed in Table 2. GAPDH was used as the housekeeping gene for loading control.

**Table 2:** Primer sequences used in this study

Gene	Primer sequence (5′-3′)	Anneal .temp. (°C)	No. of cycles	Product size (bp)
RT-PCR:				
GAPDH F	AGAAGGCTGGGGCTCATTTG	55	25	287
GAPDH R	AGGGGCCATCCACAGTCTTC			
ASC/TMS1 F	TGG GCC TGC AGG AGATG	50	36	411
ASC/TMS1 R	ATT TGG TGG GAT TGC CAG			
MSP:				
ASC/TMS1 m1	GCGGGGAGTTTAGGTTTCGTTTC	62	40	130
ASC/TMS1 m2	CCAACGCATCCAAAATAACGTCG			
ASC/TMS1 u1	GAAGGTGGGGAGTTTAGGTTTTGTTTT	62	40	140
ASC/TMS1 u2	AAATTCTCCAACACATCCAAAATAACAT			
BGS:				
ASC/TMS1 BGS F	TTG GTG TAA GTT TAGAGATAAGT	58	30	557
ASC/TMS1 BGS F	ACC ATCTCCTAC AAACCCATA			
Real Time:				
ASC/TMS1 F	TCC AGC AGC CAC TCA ACG	55	45	66
ASC/TMS1 R	GCA CTT TAT AGA CCA GCA			

### Immunohistochemistry and immunofluorescence assays

Immunohistochemical analysis of ASC/TMS1 protein expression was performed on 67 formalin-fixed, paraffin-embedded tumor tissues and paired adjacent nontumor tissues. Briefly, the sections (4-μm thickness) were deparaffinized in xylene and rehydrated by transfer through graded concentrations of ethanol to distilled water, and endogenous peroxidase activity was blocked by incubation with 3% H2O2 for 15 minutes at room temperature. Then, sections were submitted to antigen retrieval in a microwave (sodium citrate buffer, pH 6.0) for 10 minutes, naturally refrigerated to room temperature. Blocking was performed with 10% goat serum for 30 minutes at room temperature. All sections were incubated with rabbit anti-ASC/TMS1 monoclonal antibody (1:150, CST, USA), overnight at 4°C, and then incubated with goat anti-rabbit secondary antibody for 30 min at room temperature. After rinsing three times in PBS for 5 min each, the sections were incubated with DAB for 2 min, counterstained with hematoxylin for 3 min, dehydrated with gradient alcohol and transparentized with dimethylbenzene. Immunohistochemical expression of ASC/TMS1 was examined via light microscopy. Positivity was ascertained based on the presence and intensity of brown granules. All slides were reviewed independently by 2 pathologists who were blinded to each other's readings. Tissues were graded on the following scale: 0, negative; 1, weakly positive; 2, moderately positive; 3, strongly positive.

To check ASC/TMS1 protein expression after 5-Aza treatment, we performed immunofluorescence staining against ASC/TMS1 in 786–0 cells. Cells grown on glass coverslips were fixed with freshly prepared 4% paraformaldehyde for 15 min, followed by permeabilization with 0.1% Triton X-100 for 5 min on ice. Then, after washing with PBS, the cells were blocked with goat serum for 30 min. For immunofluorescence, Cells were incubated with primary antibody against ASC/TMS overnight at 4°C followed by secondary goat anti-rabbit antibody conjugated with FITC (1:200 dilution; Invitrogen, Carlsbad, CA, USA) at room temperature for 45 min. The nuclei were counterstained with DAPI (1 μg/ml; Roche, Indianapolis, IN, USA) and viewed under a fluorescence microscope.

### Methylation-specific PCR and bisulfite genomic sequencing

The bisulfite modification of purified genomic DNA was performed using an EpiTect Bisulfite Kit (Qiagen 59104, Hilden, Germany) following the manufacturer's instructions. MSP primers were examined previously for not amplifying any unbisulfited DNA and MSP products of several RCC cell lines were confirmed by direct sequencing, indicating that our MSP system was specific. For bisulfite genomic sequencing, bisulfite-treated DNA was amplified with primers specific for a fragment of the ASC/TMS1 promoter CpG islands that contained 53 CpG sites. The PCR products were subcloned into the pEasy-T5 vector (Transgene, Beijing, China) and 5–8 colonies were randomly chosen and sequenced. MSP, BGS primer sequences and PCR conditions are listed in Table 2.

### Cell viability assay

Cell viability was measured using the Cell Counting Kit-8 (Dojindo, Kumamoto, Japan) according to the instructions of the manufacturer. Briefly, A498 and 786–0 cells (2 × per well) were seeded in 96-well plates and transient transfected with PEX-2-ASC/TMS1 vector or PEX-2 empty vector. HEK293 (5 × per well) was seeded in 96-well plates and transient transfected with ASC/TMS1 siRNA or control siRNA. After transfection, at 24-hour intervals for 6 days, 10 μl of CCK-8 solution was added to each well of the plate, and the plate was incubated at 37°C for 1 hour. The absorbance was measured at 450 nm as an indicator of cell viability. All experiments were independently repeated at least 3 times.

### Colony formation analysis

Cells were plated in a 12-well plate and transfected with expression plasmid PEX-2-ASC/TMS1 or the empty vector PEX-2 (0.8 ug each) using Lipofectamine 2000. At 48 hours after transfection cells were collected and replated in a 6-well plate and selected for 2 weeks with G418 (0.4 mg/ml). Surviving colonies (50 or greater cells per colony) were counted after staining with crystal violet. All experiments were independently repeated at least 3 times.

### Wound-healing assay

Cell migration was assessed using a scratch wound assay. Briefly, PEX-2-ASC/TMS1 or empty vector stably transfected 786–0 cells were cultured in six-well plates. When the cells grew up to 80–90% confluence, a wound was made by dragging a plastic pipette tip across the cell surface. The phase contrast images of the wounds were recorded at 37°C for incubations of 0, 12, 24, 36 hours, and 3 separate experiments were performed.

### Cell invasion assay

Cell invasion assays were performed using 24-well transwells (8-μm pore size; BD Biosciences), coated with Matrigel (Falcon354480; BD Biosciences). RCC cells were starved overnight in serum-free medium, trypsinized, and washed 3 times in DMEM containing 1% FBS. A total of 1 × 105 cells was then suspended in 500 μl DMEM containing 1% FBS and added to the upper chamber, while 750 μl DMEM containing 10% FBS was placed in the lower chamber. For the control, medium containing 1% FBS was added to the lower chamber. After 24 hours of incubation, Matrigel and cells remaining in the upper chamber were removed by cotton swabs. Cells on the lower surface of the membrane were fixed in 4% paraformaldehyde and stained with 0.5% crystal violet. Cells in at least 6 random microscopic fields (magnification, × 100) were counted and photographed. All experiments were repeated 3 times.

### Flow cytometry

The stably transfected 786–0 cells with PEX-2-ASC/TMS1-expressing or PEX-2- and A498 ASC/TMS1 empty vector were fixed in 70% ethanol and stained with 50 mg/ml propidium iodide (BD Pharmingen, San Jose, CA, USA). The cells were then sorted by FACSCalibur (BD Biosciences, Franklin Lakes, NJ, USA) and cell cycle profiles were analyzed by ModFit 3.0 software (Verity Software House, Topsham, ME, USA). Apoptosis was assessed using the dual staining with Annexin V:PE (BD Biosciences) and 7-amino-actinomycin (BD Biosciences). Briefly, PEX-2-ASC/TMS1 or empty vector-transfected cells were harvested at 48 h post transfection. Annexin V:PE and 7-amino-actinomycin were added to the cellular suspension according to the manufacturer's instructions and was analyzed by flow cytometry (FACSCalibur, BD, Franklin Lakes, NJ, USA). Both early and late apoptotic cells were counted for relative apoptotic changes. All the experiments were performed at least three times.

### Western blot analysis

Total protein was extracted with RIPA (Radio-Immunoprecipitation Assay) lysis buffer, supplemented with 1mM Na3VO4, 1mM NaF, 1X protease inhibitor and 1mMPMSF before use. Equal amounts of proteins were separated by 10–12% SDS-PAGE gel and then transferred onto nitrocellulose membranes. After blocking the membranes with 5% fat free milk in TBST for 1 h at room temperature, the membranes were incubated with specific primary antibodies overnight at 4°C. After washing, the membrane was incubated with HRP-conjugated secondary antibodies for 1 h at room temperature and then washed with TBST. The antibody complexes in the immunoblots were detected by chemiluminescence using an HRP substrate (Millipore, Bedford, MA, USA; WBKLS0100) and visualized using a G:BOX Chemi Gel Documentation System (Syngene, Frederick, MD, USA).

### Knockdown of ASC/TMS1 by siRNA

Cells were transfected with a control non-targeting siRNA or with an ASC/TMS1-targeting siRNA (50 nM) using Lipofectamine 2000 according to the manufacturer's instructions. For DNA damaging agent treatment, after 48 h of transfection, Caki-2 cells were treated with etoposide (50 uM) or doxorubicin (1 uM) and analyzed by Western blot for the indicated antibodies.

### *In vivo* tumorigenicity

786–0 cells (1 × 10^6^ cells in 0.2 ml phosphate-buffered saline) stably transfected with PEX-2-ASC/TMS1 vector or PEX-2 empty vector were injected subcutaneously into the dorsal right flank of 4-week-old male SCID mice, separately. Tumor diameter was measured every 3 days for 18 days. Tumor volume (mm^3^) was estimated by measuring the longest and shortest diameter of the tumor and calculating as follows: volume = (shortest diameter)^2^ × (longest diameter) × 0.5^5^ as described previously [46]. All mice were maintained in accordance with the NIH Guidelines for the Care and Use of Laboratory Animals with the approval of Review Board of Peking University First Hospital, Beijing.

### Statistical analysis

All values were expressed as mean ± standard deviation (SD) of n observations. The significance of differences between two groups was determined using a two-tailed *t*-test. The Fisher exact test or chi-square test was used for analysis of patient features. The difference in tumor growth rate between the two groups of mice was determined by repeated-measures analysis of variance. *P* < 0.05 was considered statistically significant.
